# Species-Specific Expansion and Molecular Evolution of the 3-hydroxy-3-methylglutaryl Coenzyme A Reductase (HMGR) Gene Family in Plants

**DOI:** 10.1371/journal.pone.0094172

**Published:** 2014-04-10

**Authors:** Wei Li, Wei Liu, Hengling Wei, Qiuling He, Jinhong Chen, Baohong Zhang, Shuijin Zhu

**Affiliations:** 1 Department of Agronomy, Zhejiang University, Hangzhou, Zhejiang, China; 2 State Key Laboratory of Cotton Biology, Cotton Research Institute, Chinese Academy of Agricultural Sciences, Anyang, Henan, China; 3 Department of Biology, East Carolina University, Greenville, North Carolina, United States of America; Nanjing Agricultural University, China

## Abstract

The terpene compounds represent the largest and most diverse class of plant secondary metabolites which are important in plant growth and development. The 3-hydroxy-3-methylglutaryl coenzyme A reductase (HMGR; EC 1.1.1.34) is one of the key enzymes contributed to terpene biosynthesis. To better understand the basic characteristics and evolutionary history of the HMGR gene family in plants, a genome-wide analysis of HMGR genes from 20 representative species was carried out. A total of 56 HMGR genes in the 14 land plant genomes were identified, but no genes were found in all 6 algal genomes. The gene structure and protein architecture of all plant HMGR genes were highly conserved. The phylogenetic analysis revealed that the plant HMGRs were derived from one ancestor gene and finally developed into four distinct groups, two in the monocot plants and two in dicot plants. Species-specific gene duplications, caused mainly by segmental duplication, led to the limited expansion of HMGR genes in *Zea mays*, *Gossypium raimondii*, *Populus trichocarpa* and *Glycine max* after the species diverged. The analysis of Ka/Ks ratios and expression profiles indicated that functional divergence after the gene duplications was restricted. The results suggested that the function and evolution of HMGR gene family were dramatically conserved throughout the plant kingdom.

## Introduction

Plants produce thousands of secondary metabolites that play important roles in numerous biological processes. Structurally and functionally different terpenes represent the largest and most diverse class of secondary metabolites [1]. In addition to their physiological functions in photosynthesis, respiration, and growth and development, many specialized terpenes also have ecological roles in mediating plant interactions with various biotic and abiotic factors [2]. For example, terpenes can serve as phytoalexins in defense against phytopathogens and herbivores [3], [4], [5], [6], and low-molecular-weight terpene compounds may released as odors that attract pollinators or induce defense responses in neighboring plants [7], [8].

In plant cells, two distinct pathways are responsible for the biosynthesis of terpene compounds, the cytosolic mevalonate pathway (MVA pathway) and the plastidial 2-C-methyl-D-erythritol-4-phosphate pathway (MEP pathway) [9]. The reaction catalyzed by the enzyme 3-hydroxy-3-methylglutaryl coenzyme A reductase (HMGR) is the first committed step in the MVA pathway [10]. The gene encoding HMGR has been found that widely present in eukaryotes and prokaryotes, and well studied in mammals due to its critical role in mediating cholesterol biosynthesis [11]. In plants, the HMGR genes also have been extensively cloned and characterized from a number of species including *Arabidopsis thaliana* [12], [13], *Oryza sativa* [14], *Triticum aestivum* [15], *Gossypium hirsutum* [16], *Solanum tuberosum* [17], *Cucumis melo* [18], *Hevea brasiliensis* [19] and some medicinal plants [20], [21], [22]. Additionally, HMGR is considered as a key enzyme for biotechnological purposes and can be utilized to increase terpenes content in plants. As a result, up-regulation of HMGR genes could improve terpenes productivities in the transgenic plants [23], [24], [25], [26], [27]. Furthermore, it was reported that transgenic tomato plants that constitutively expressed a heterologous HMGR gene from melon showed a significant increase in fruit size [28].

HMGR protein is comprised of three domains, i.e., the transmembrane domains with changeable number in N-terminal region, the highly divergent linker domain, and the long and conserved catalytic domain in C-terminus. Within the catalytic domain, subdomains have been defined as the small helical N-terminal domain, the large and central L-domain harboring two HMG-CoA binding motifs and an NADP(H) binding motif, and the smallest S-domain harboring an NADP(H) binding motif. The two HMG-CoA binding motifs, EMPVGYVQIP and TTEGCLVA, and two NADP(H) binding motifs, DAMGMNM and GTVGGGT, are functionally important and thus highly conserved in all HMGR proteins [29], [30].

Although HMGR genes have been systematically analyzed in Gramineae plants [30], little is known about their features in the genome-wide level in plant kingdom. Thus, a comprehensive investigation about the basic characteristics and evolutionary history of this gene family is necessary in plants. Fortunately, the recent availability of whole genome sequences of various plant species in public databases offers an opportunity to identify the complete set of HMGR genes in many species. In present study, an extensive survey of HMGR families in 20 species ranging from unicellular algae to higher plants was conducted. Subsequently, the distribution, protein architecture, exon/intron organization, phylogenetic relationship and expansion pattern of this gene family were assessed, and the expression profiles of HMGR genes in *Zea mays* and *Glycine max* using published transcriptome data were analyzed as well.

## Materials and Methods

### Identification and verification of HMGRs in different plant genomes

The amino acid sequences of *Arabidopsis thaliana* HMGR genes [2] were retrieved from The Arabidopsis Information Resource (TAIR, http://www.arabidopsis.org) and used as queries to search against other plant genome databases with BlastP and tBlastN programs (default parameters). The 20 completely sequenced genomes of species from unicellular green algae to multicellular higher plants were used in this study (Table 1 and Table S1). Subsequently, all hits considered as candidate sequences were submitted to Pfam database (http://pfam.sanger.ac.uk/) to confirm the presence of the conserved domain (PF00368).

**Table 1:** Species and number of HMGR genes used in this study.

Lineage	Species	Abbreviation	Number of HMGR genes
Green algae	*Ostreococcus lucimarinus*	Ol	0
	*Ostreococcus tauri*	Ot	0
	*Micromonas pusilla*	Mp	0
	*Chlamydomonas reinhardtii*	Cr	0
	*Chlorella variabilis*	Cv	0
	*Volvox carteri*	Vc	0
Mosses	*Physcomitrella patens*	Pp	3
Lycophytes	*Selaginella moellendorffii*	Sm	1
monocots	*Brachypodium distachyon*	Bd	3
	*Oryza sativa*	Os	3
	*Zea mays*	Zm	7
	*Sorghum bicolor*	Sb	3
Eudicots	*Vitis vinifera*	Vv	3
	*Citrus sinensis*	Cs	2
	*Gossypium raimondii*	Gr	9
	*Carica papaya*	Cp	3
	*Brassica rapa*	Br	3
	*Arabidopsis thaliana*	At	2
	*Glycine max*	Gm	8
	*Populus trichocarpa*	Pt	6
Total			56

### Protein motif and gene structure analysis

The conserved motifs encoded by each HMGR gene were identified using the program of Multiple Em for Motif Elicitation (MEME; version 4.9.0) [31] at the website (http://meme.nbcr.net/meme/cgi-bin/meme.cgi). The analysis was performed with a set of parameters as follows: number of repetitions, any; minimum width for each motif, 6; maximum width for each motif, 100; and maximum number of motifs to be found, 5. All obtained motifs were searched in the InterPro database with InterProScan [32]. The exon/intron structures of HMGR genes were obtained by comparing the genomic sequences and their predicted coding sequences (CDS) using GSDS (http://gsds.cbi.pku.edu.cn/) [33].

### Multiple sequence alignment and phylogenetic reconstruction

Multiple sequence alignments of the full-length protein sequences were performed by Clustal X version 2.0 program [34] with default parameters. The neighbor-joining (NJ) phylogenetic tree was constructed using MEGA 5.2 [35] with pairwise deletion option. The reliability of obtained phylogenetic tree was tested using bootstrapping with 1000 replicates.

### Chromosomal mapping and gene duplications

The locations of HMGR genes in *Zea mays*, *Gossypium raimondii*, *Populus trichocarpa* and *Glycine max* were collected from the genome annotation data of the corresponding organism, respectively. The chromosomal distribution images of these HMGR genes were generated by MapInspect software according to their starting positions in chromosomes [36]. Gene duplication events of HMGR genes in *Zea mays*, *Gossypium raimondii*, *Populus trichocarpa* and *Glycine max* were also investigated. Gene duplication was defined according to (1) the length of aligned sequence cover >80% of the longer gene; and (2) the identity of the aligned regions >80% [37], [38]. With the chromosomal locations of HMGR genes, two types of gene duplications were recognized, i.e., tandem duplication and segmental duplication.

### Estimating the divergence time for duplicated gene pairs

The pairwise alignment of HMGR duplicated gene pairs from four plants was performed using Clustal X version 2.0 program [34]. Then pairwise synonymous (Ks) and non-synonymous (Ka) numbers of substitutions corrected for multiple hits were calculated using the DnaSP v5.0 software (DNA polymorphism analysis) [39]. Finally, the selection pressure for these duplicate HMGR gene pairs was calculated as Ka/Ks ratio. Based on the synonymous substitutions per year (λ) of 6.5×10^−9^ for *Zea mays* [40], 9.1×10^−9^ for *Populus trichocarpa* [41], and 6.1×10^−9^ for *Glycine max* [42], by substituting the calculated Ks values, the approximate age of duplicated events of the duplicate HMGR gene pairs was estimated (T =  Ks/2λ×10^−6^ Mya).

### Gene expression analysis

The expression profiles of ZmHMGRs and GrHMGRs were clustered using the Cluster 3.0 [43], respectively. The public expression data for various tissues and developmental stages in *Zea mays* were obtained from the Plant Expression Database (PLEXdb, http://www.plexdb.org/) [44], [45] according to the identified ZmHMGR ID, and the transcriptome sequencing datasets of *Glycine max* were downloaded from SoyBase (http://soybase.org/soyseq/) [46] based on the GmHMGR ID.

## Results and Discussion

### Genomic identification of HMGR genes in plants

In order to identify HMGR genes in Viridiplantae, the blast searches among the 20 completely sequenced genomes (Table S1) were carried out. These genomes represent major evolutionary lineages of the plant kingdom such as algae, mosses, lycophytes, monocots, and eudicots. After removing partial or redundant sequences, and the predicted alternative splice variants, a total of 56 genes encoding HMGR proteins were retrieved in the 14 land plant genomes, and no HMGR genes were detected in algae (Table 1). Because there is no standard naming system for HMGR genes (not including Arabidopsis), the newly identified HMGR genes were assigned according to the species and the gene orders on the chromosomes (Table S2). The Arabidopsis HMGR genes were named following the TAIR website (http://www.arabidopsis.org/).

The Viridiplantae are comprised of two major lineages that split early, i.e., the Chlorophyta (chlorophyte algae) and the Streptophyta (charophyte algae and land plants) [47], [48]. Interestingly, it was observed that no HMGR genes were found in genomes of all 6 algal species, which belong to the division Chlorophyta of green algae, suggesting that the HMGR gene family might be lost in the chlorophyte algae during evolution. The HMGR protein is a major rate-limiting enzyme in the MVA pathways [10], and the MVA pathway is considered as an ancestral metabolic route for the biosynthesis of terpene compounds in all the three domains of life (bacteria, archaea, and eukaryotes) [49]. So it could be speculated that the Chlorophyta had abandoned the MVA pathway. These observations were compatible with previous findings that the chlorophyte algae synthesized their terpenes exclusively via the MEP pathway and might develop efficient mechanisms of exporting MEP-pathway-derived terpene intermediates from the plastid for the biosynthesis of cytosolic terpenes [50], [51]. The analysis also provided further proof for that the genes involved in the MVA pathway in the chlorophyte algae were not silenced but really absent.

Although genome sequence data are currently not available for charophyte algae, which is believed to be the closest relatives of land plants [47]. But it has been experimentally substantiated that the charophyte algae *Mesostigma viride* contained the HMGR gene [52]. Moreover, it was also found that the HMGR genes were widespread in land plants. So it could be figured out that the MVA pathway was still operating in the Streptophyta, especially in land plants. The MEP pathway has been identified to be present in plastid-bearing eukaryotes [53]. Therefore, unlike the chlorophyte algae, the land plants simultaneously retained the MVA and MEP pathways. In this case, the MVA pathway was active within the cytoplasm at the same time as the MEP pathway functioned in the plastids. The utilization of both the MVA and MEP pathways could enable plants to separate and optimize the biosynthesis of a wide range of complex terpene-derived specialized metabolites. Obviously, by retaining and compartmentalizing the two pathways, the land plants could gain a selective advantage in interactions with their surrounding environments to overcome sessile-lifestyle constraints.

Additionally, among the land plants, there were 1 to 9 HMGR genes in each species, and most species (10 of 14 species) only had 3 or less HMGR genes. The non-vascular *Physcomitrella patens*, a species of mosses which is a basal lineage of land plants, contained 3 HMGR genes. The *Selaginella moellendorffii*, the oldest extant vascular plant belonging to lycophytes, was the fewest HMGR gene family species among the land plants in our survey, which was expected as it has one of the smallest plant genomes known [54]. In the flowering plants, the number of HMGR genes was varied greatly. Here, *Citrus sinensis* and *Arabidopsis thaliana* contained only two HMGR genes, which was the fewest two species in HMGR gene number among flowering plants we investigated. The other six plants, *Brachypodium distachyon*, *Oryza sativa*, *Sorghum bicolor*, *Vitis vinifera*, *Carica papaya*, and *Brassica rapa*, had three HMGR gene members. While the remaining four species had a relative higher number of HMGR genes, 6 HMGRs in *Populus trichocarpa*, 7 HMGRs in *Zea mays*, 8 HMGRs in *Glycine max*, and 9 HMGRs in *Gossypium raimondii*. The variable size of the HMGR gene family suggested that the gene family underwent species-specific expansion in flowering plants. Furthermore, among these species which underwent the gene expansion, *Zea mays* belongs to the monocots, and the other three belong to the eudicots, which indicated that the gene expansion occurred both in monocot plants and dicot plants.

### Conserved protein motifs and exon/intron structure of HMGR genes

**Figure 1:**
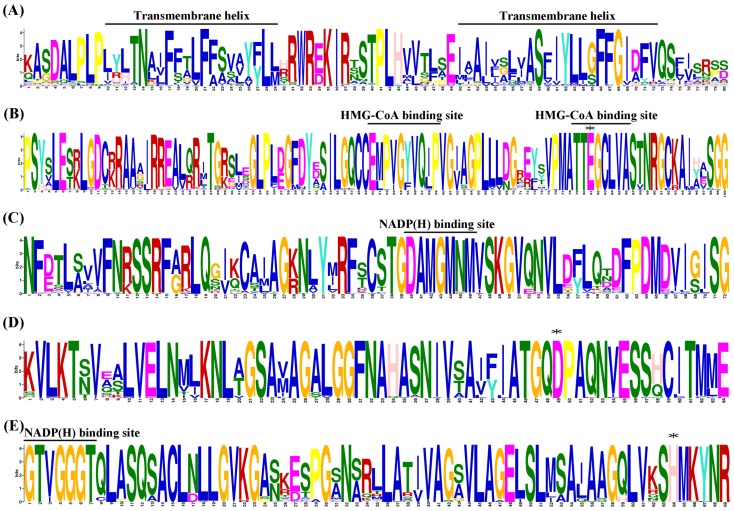
Sequence logos of the five motifs identified using the MEME search tool. (A), (B), (C), (D) and (E) represent the motif 5, 3, 2, 1 and 4, respectively. The height of letter designating the amino acid residue at each position represents the degree of conservation. The numbers on the x-axis represent the residue positions in the motifs. The y-axis represents the information content measured in bits. The two transmembrane helices, two HMG-CoA binding sites (EMPVGYVQIP and TTEGCLVA) and two NADP(H) binding sites (DAMGMNM and GTVGGGT) are represented on the top of the corresponding locations in motifs. Asterisks (*) indicate the conserved residues in the catalytic domain of plant HMGR genes.

The MEME motif search tool was employed to identify the conserved motifs presented in 56 plant HMGR proteins, and 5 conserved motifs were uncovered (Figure S1). After searching in the Interpro database, all motifs corresponded to known domains. The motif 5 was a region including two transmembrane helices, and the others were located in the catalytic domain of HMGR genes (Figure 1).

A common feature of plant HMGRs is the presence of a transmemebrane region consisting of two separate transmemebrane domains that are linked to the cytoplasmic domain bearing the catalytic center [55], [56]. Of the 56 HMGRs, only VvHMGR3, GrHMGR8 and GmHMGR3 missed the motif 5, suggesting that most plant HMGR genes have two transmembrane helices in the N-terminus. The result was verified by prediction of transmembrane helices in HMGR proteins using the TMHMM Server v. 2.0 (Table S2). Unexpectedly, existence of the motif 5 and the prediction of two transmembrane helices by TMHMM Server v. 2.0 did not correspond precisely in five HMGR genes. Further analysis found that there were no transmembrane helices that could be predicted by TMHMM Server v. 2.0 due to the diversity of several amino acid sites in the motif 5 in these genes. Overall, the plant HMGRs usually had two transmemebrane domains in the N-terminal region of proteins, and several genes appeared to start sequence variations, or even lost the domain.

In the protein sequences of plant HMGR, there were four conserved motifs including EMPVGYVQIP, TTEGCLVA, DAMGMNM, and GTVGGGT in the catalytic domain. In this study, the motif 3 represented the two HMG-CoA binding sites (EMPVGYVQIP and TTEGCLVA), the motif 2 represented one of the NADP(H) binding sites (DAMGMNM), and the motif 4 represented the other NADP(H) binding sites (GTVGGGT), respectively (Figure 1). Among them, the motif 3 in the N-terminus was lost in GrHMGR8 and GmHMGR3, the motif 2 in the middle was lost in OsHMGR1 and ZmHMGR1, and the motif 4 in the C-terminus was lost in OsHMGR1, ZmHMGR1, ZmHMGR3, ZmHMGR5 and GmHMGR3 (Figure S1). Apparently, the C-terminus of the catalytic domain was more variable than the N-terminus in the evolutionary process of plant HMGRs. Interestingly, 3 out of 7 ZmHMGRs missed the motif 4 that binds to NADP(H), and the ZmHMGR1 also missed the other NADP(H) binding sites (motif 2). It could be guessed that this might be the need for functional differentiation of HMGR genes after gene expansions in maize.

In order to validate the conservation of residues in the catalytic domain, the sequence logos of the motif 3, motif 2, motif 1 and motif 4 were investigated (Figure 1). It was observed that the high homology region appeared to be centered around the HMG-CoA and NADP(H) binding sites. The amino acids composed of the second HMG-CoA binding site and the two NADP(H) binding sites were almost the same in all analyzed plant HMGRs. While the amino acid residues in the first HMG-CoA binding site were diverse among plant HMGRs, which might contribute to the substrate selectivity. Additionally, the position and orientation of four key catalytic residues (Glu, Lys, Asp and His) [29], which are functionally significant, were highly conserved in HMGR proteins. Among them, three residues except Lys residue were shown in Figure 1.

Analysis of HMGR gene structure for exon/intron organization revealed that the number of introns per gene varied from 1 to 14 (Figure S2). OsHMGR1, ZmHMGR1, and ZmHMGR5 possessed a minimum of one intron each, whereas ZmHMGR3 possessed a maximum of fourteen introns. Among all the analyzed genes, the great majority (43 out 56), including all HMGRs from the lower land plants, possessed three introns. These results indicated that the common ancestor of plant HMGRs had three introns. The intron/exon structure of HMGR genes was highly coincided in plant evolution.

Regardless of the less variability, the protein architectures and gene structures of plant HMGRs were remarkably conserved, indicating that the molecular characteristics and biological functions of each plant HMGR were highly conserved during evolution. This was not the same as other gene families involved in plant secondary metabolism, which were very much lineage-dependent and varied tremendously among plant taxa [57]. The results also suggested that the plant HMGR genes might be monophyletic and were descendants of an ancestor.

### Phylogenetic analysis of HMGR gene families

**Figure 2:**
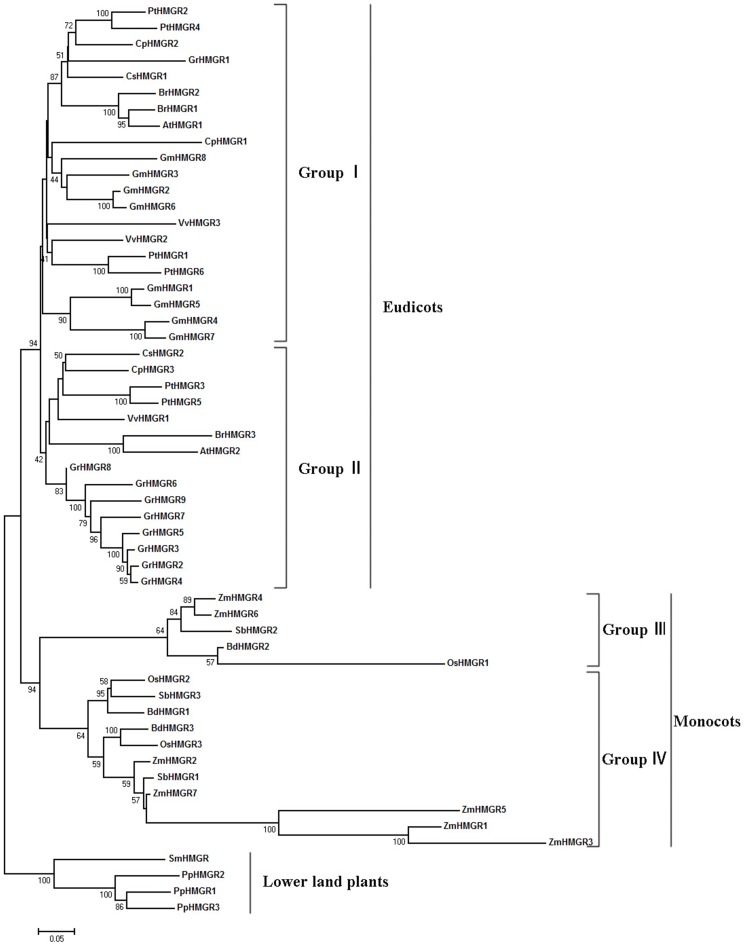
Neighbor-joining phylogenetic tree of plant HMGR proteins. The tree was constructed based on a complete protein sequence alignment of HMGR genes using neighbor-joining method, and the PpHMGRs and SmHMGR were designated as outgroups. Numbers at the nodes represent bootstrap support (1000 replicates). Bootstrap support higher than 40% is indicated at respective nodes.

To examine the evolutionary relationships of the HMGR genes in Viridiplantae, a neighbor-joining phylogenetic tree (Figure 2) was constructed based on the alignments of full-length HMGR protein sequences. Because mosses and lycophytes are the basal lineages of land plants, PpHMGRs and SmHMGR were designated as outgroups. Firstly, HMGR genes from the flowering plants were divided into two monophyletic clades, the monocots and eudicots. No genes from the two lineages tended to cluster together in the phylogenetic tree, suggesting that the plant HMGRs were derived from one ancestor gene and developed into different branches after the lineages diverged. Within the monocots or eudicots, the HMGR genes fell into two major distinctive groups each with high bootstrap values. As shown in the Figure 2, these groups were named group I, II, III and IV.

In eudicots, there were 36 HMGR genes from 8 plant species. Group I contained 21 members, and group II contained 15 members. Statistically, 7 out 8 dicot plants contained all two groups of HMGR genes, except for *Glycine max*, which only contained group I HMGR genes. Obviously, the significant expansion of group I genes contributed to the increase of HMGR genes in *Glycine max* which had up to 8 members. In addition, those HMGR genes from the same species were not equally classified into the two groups. Most of the 8 plant species had more genes in group I than in group II, except for *Gossypium raimondii* which contained 8 group II genes, but only a single gene in group I. It was indicated that, unlike *Glycine max*, the increase in HMGR genes in *Gossypium raimondii* attributed to the remarkable expansion of group II genes.

In monocots, all of 4 monocot plants contained the two group genes, group III and IV. As the HMGR genes from dicot plants, the 16 HMGR genes from 4 monocot plants were not evenly distributed in the two groups neither. All investigated monocot plants contained more members in group IV than in group III. Such as *Zea mays* that had the largest HMGR gene family in analyzed monocots had five genes in group IV, but two genes in group III.

In general, there were no paralogs of HMGR genes from plants which contained less than or equal to 3 HMGRs, indicating that no gene expansion occurred within the HMGR gene family after the divergence of these plant species. In contrast, four other species, *Zea mays*, *Gossypium raimondii*, *Populus trichocarpa*, and *Glycine max*, underwent considerably more frequent gene duplications, which gave rise to an increase in more members of the HMGR gene family. As we know, the occurrence of most of secondary metabolites and their respective biosynthetic pathways is restricted to specific plants or plant lineages [57]. The evolution of these pathways definitely requires new enzymes and regulatory elements in specific plants. So it can be deduced that the four plant species may need to produce more or wider variety of terpene compounds in their respective development. *Gossypium raimondii*, which contained the largest HMGR gene family (9 genes) in our survey, was known to accumulate a unique group of terpenes included desoxyhemigossypol, hemigossypol, gossypol, hemigossypolone, and the heliocides [58], which fit well with this hypothesis.

### Chromosomal localization and duplication of HMGR genes in four selected plant species

**Figure 3:**
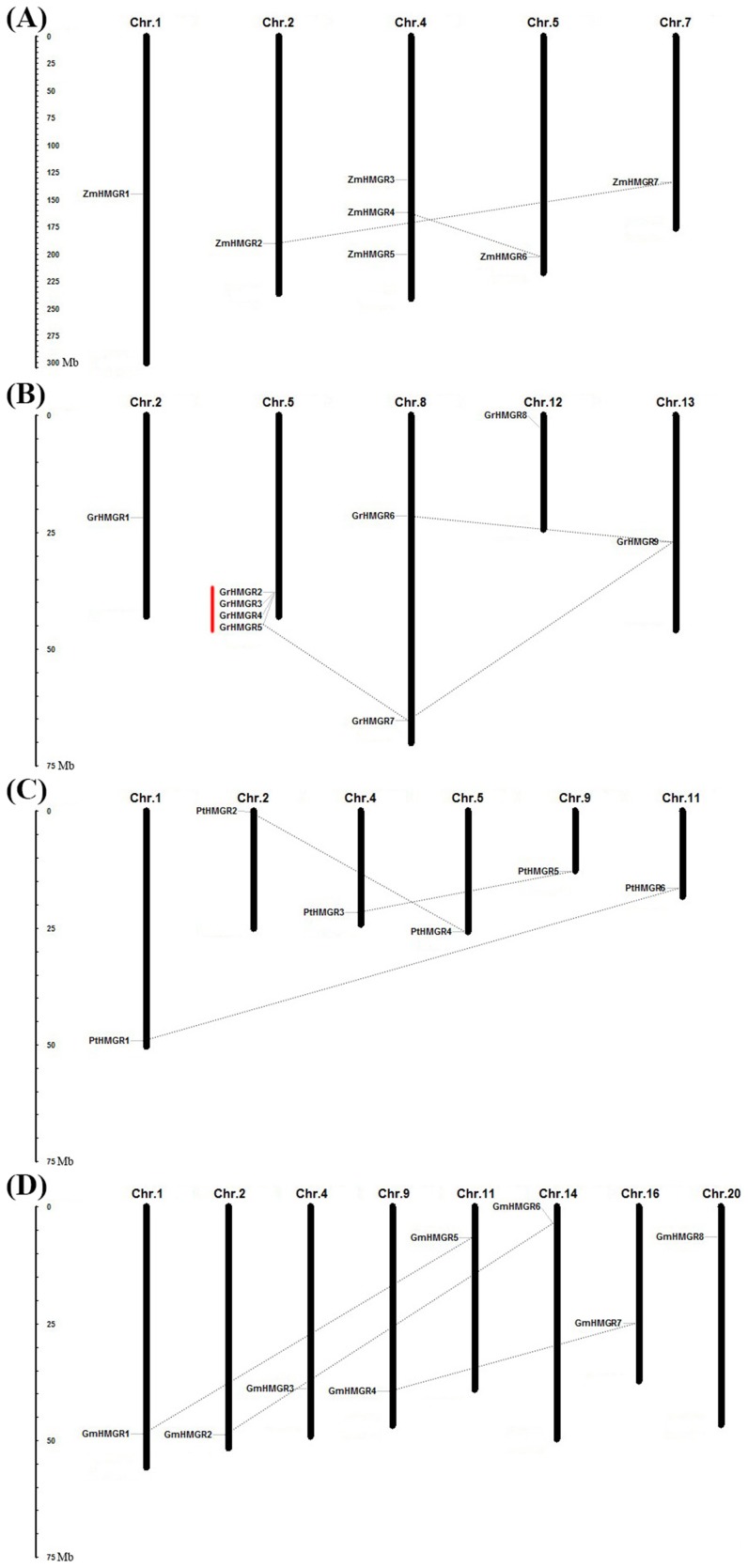
Chromosomal localization of HMGR genes in four selected plant species. (A): *Zea mays*; (B): *Gossypium raimoddii*; (C): *Populus trichocarpa*; and (D): *Glycine max*. The chromosome number is indicated at the top of each chromosome representation. The scale represents megabases (Mb). The segmental duplicated genes are indicated by dotted lines and the tandemly duplicated genes by red vertical lines.

Based on the coordinate of each HMGR gene on the chromosomes, the chromosomal distribution images of these HMGR genes in four plant species which underwent species-specific gene expansions were generated (Figure 3). The 7 HMGR genes of *Zea mays* were distributed unevenly on 5 chromosomes, and chromosome 4 contained three HMGR genes. In *Gossypium raimondii*, 9 HMGR genes were located on 5 chromosomes, and one gene cluster contained 4 genes was detected on chromosome 5. While in *Populus trichocarpa* and *Glycine max*, the HMGR genes were distributed uniformly, one gene on each chromosome.

A total of fourteen duplication events which contributed to HMGR gene family expansion in the four plant species were found. Among them, two events as segmental duplications were in *Zea mays*, and there were three segmental duplication events in respective genome of *Populus trichocarpa* and *Glycine max*, strongly indicating that the segmental duplication was the main cause of the species-specific expansion of HMGR genes in the three species. Particularly, there were up to six duplication events in *Gossypium raimondii* which had the largest HMGR gene family number in our survey. With three segmental duplications and three tandem duplications, it was suggested that a relatively large extent of species-specific gene expansion was caused by both segmental and tandem duplications after the divergence of *Gossypium raimondii*.

During the process of evolution, the duplicated genes might have undergone divergent fates such as nonfunctionalization (loss of original functions), neofunctionalization (acquisition of novel functions), or subfunctionalization (partition of original functions) [41]. To explore whether Darwinian positive selection was involved in HMGR gene divergence after duplication, the Ka/Ks ratio for each pair of duplicated HMGR genes were calculated (Table S3). Generally, a Ka/Ks ratio >1 indicates accelerated evolution with positive selection, a ratio  =  1 indicates that the genes are pseudogenes with neutral selection, while a ratio <1 indicates the functional constraint with negative or purifying selection of the genes. In this study, the Ka/Ks ratios for fourteen duplicated HMGR gene pairs were less than 0.3, suggesting that the HMGR genes from the four plants have mainly experienced strong purifying selection pressure with limited functional divergence after the species-specific duplications. These results suggested that functions of the duplicated HMGR genes did not diverge much during subsequent evolution. The approximate age of segmentally duplicated HMGR gene pairs from *Zea mays*, *Populus trichocarpa* and *Glycine max* were estimated using the Ks as the proxy for time (Table S3). The Ks values of these duplicated HMGR gene pairs were 0.152–0.289, indicating that the duplications might have occurred 12.25–16.05 million years ago (Mya).

### Expression profiling of HMGR genes in Zea mays and Glycine max

To understand the temporal and spatial expression patterns of HMGR genes, their expression profiles during *Zea mays* and *Glycine max* development were analyzed using the public expression data.

**Figure 4:**
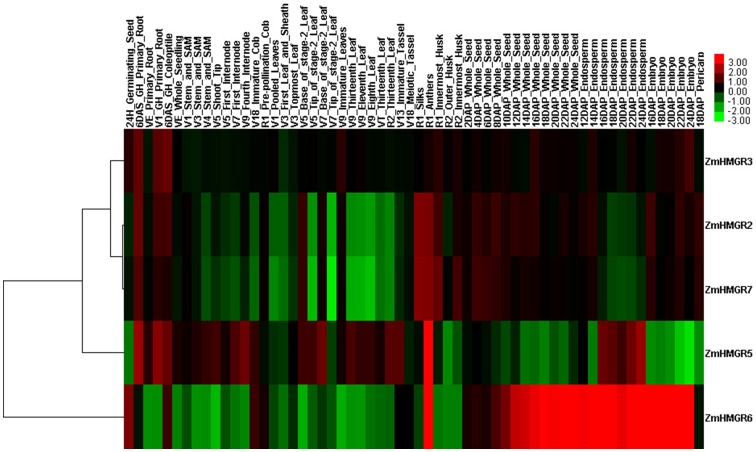
Expression profiles of HMGR genes in *Zea mays* across different tissues and developmental stages. The color scale represents the relative signal intensity values. DAP: Days After Pollination; DAS: Days After Sowing.

The published microarray data of 60 different tissues and developmental stages of *Zea mays* [44] were collected and investigated (Figure 4). Five of the seven ZmHMGRs showed wide expressions in the examined tissues. The ZmHMG6 showed higher expression in seeds (whole seed, endosperm and embryo) than in other organs, which indicated that it may play important roles in seed development or secondary metabolites accumulation in maize seed. By contrast, the ZmHMGR5 just expressed specificity in the endosperm, but not the whole seed and embryo. Additionally, the ZmHMGR5 showed relatively high expression in vegetative tissues (root, internode and leaf). The ZmHMGR2, ZmHMGR3 and ZmHMGR5 also showed remarkable expression in roots. In the anthers, there were four ZmHMGRs (ZmHMGR2, ZmHMGR5, ZmHMGR6 and ZmHMGR7) expressed highly, which might be due to the large demand for terpene compounds in the pollen development [59]. However, two ZmHMGRs (ZmHMGR1 and ZmHMGR4) were not found to have corresponding probes in this dataset, so there were no expression data to be investgated. As the ZmHMGR4 had the typical gene structure (3 introns) and was just diversified in the transmemebrane region of the N-terminus in protein sequence, and it also was involved in a duplicated event with ZmHMGR6 that was expressed widely in maize development. It was deduced that the ZmHMGR4 might undergo the process of subfunctionalization after gene duplication and might be expressed similarly with ZmHMGR6. The other ZmHMGR duplicated gene pair, ZmHMGR2 and ZmHMGR7, which had the same gene structure and protein architecture shared similar expression patterns in nearly all of the organs and developmental conditions analyzed.

**Figure 5:**
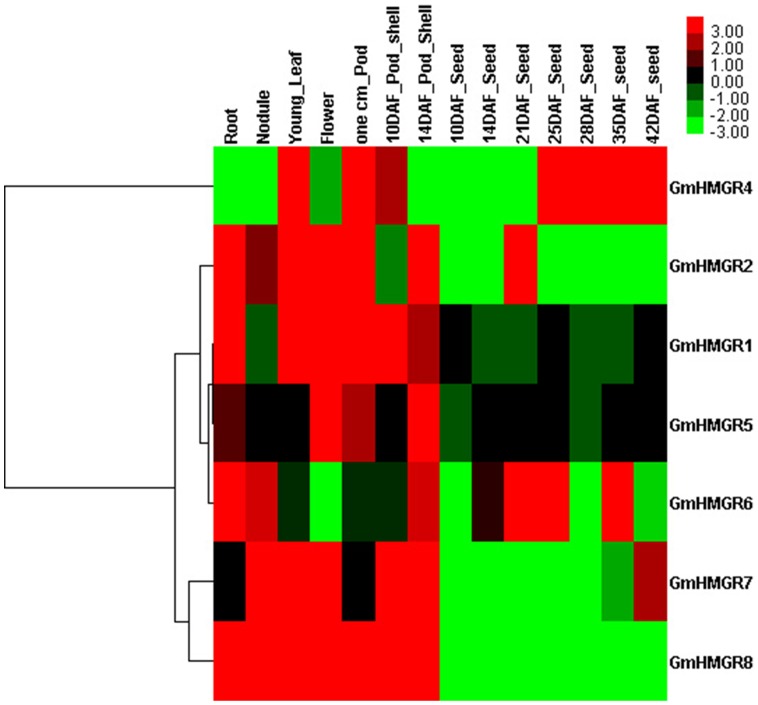
Expression profiles of HMGR genes in *Glycine max* across different tissues and developmental stages. The color scale represents the relative signal intensity values. DAF: Days After Flowering.

The expression profiles of HMGR genes in *Glycine max* [46] were also analyzed (Figure 5). Similar to ZmHMGRs, the GmHMGRs also exhibited abundant transcript across multiple tissues and organs. Moreover, there was also a HMGR gene (GmHMGR4) expressed at relatively high level in the seeds. Especially, it had higher expression in later developmental stages but lower expression in early developmental stages of seed, which suggested that it might contribute to the accumulation of terpene compounds in soybean seed. Additionally, the expression patterns of all identified soybean duplicated gene pairs which had the same gene structure and protein architecture were also investigated in this research. Most gene pairs such as GmHMGR1/GmHMGR5 and GmHMGR2/GmMGR6 were similar. However, it was not the case for GmHMGR4/GmHMGR7. The expression of the duplicated gene pairs were strongly divergent, which might be caused by the significant variation in gene regulations after the duplication events. Overall, the highly similar expression patterns of most duplicated gene pairs in *Zea mays* and *Glycine max* implied that functional divergence after the gene duplications was restricted. These results indicated that the HMGR gene family was dramatically conserved during plant genome evolution.

## Conclusions

The HMGR gene family might be lost in the chlorophyte algae during evolution, but still widespread in land plants. The plant HMGR genes might be derived from one ancestor gene and finally developed into two distinct groups within the monocots and eudicots, respectively. The gene structure and protein architecture of all plant HMGRs were considerable conserved. The HMGR family in four flowering plants underwent species-specific expansions after the species diverged due to the large production for terpene compounds in their respective development. Segmental duplication appeared to be the dominant mechanism for the gene duplication events in three species, whereas segmental duplication and tandem duplication played similar roles in the expansion of the HMGR gene family of *Gossypium raimondii*. The functional divergence after the gene duplications was restricted. The findings implied that the HMGR gene family was dramatically conserved during plant evolution, and the HMGR was the committed enzyme for terpene biosynthesis that had essential roles in regulating plant development.

## Supporting Information

Figure S1 **Distribution of conserved motifs in plant HMGR proteins identified using the MEME search tool.** Different motifs are indicated by different colors numbered 1-5. The names of all members of HMGR genes and combined *p*-values are shown on the left side of the figure, and the positions and sizes of motifs are indicated at the bottom of the figure. The motif 5 is a region including two transmembrane helices. The motif 3, 2, 1 and 4 are located in the catalytic domain of HMGR proteins. Moreover, the N-terminus of the motif 3 is at the start position of catalytic domain in each HMGR protein.
(TIF)

Figure S2 **Exon/intron organization of plant HMGR genes.** Exons are represented by green boxes and introns by black lines. Names of all plant HMGR genes from different lineages are shown on the left side of the figure.
(TIF)

Table S1 **Data sources for the genome sequences used to mine for HMGR genes.**
(XLSX)

Table S2 **Summary of 56 HMGR genes identified in Viridiplantae.**
(XLSX)

Table S3 **Ka/Ks analysis and estimated divergence time for the HMGR duplicated genes from** ***Zea mays*** **,** ***Gossypium raimondii*** **,** ***Populus trichocarpa*** **and** ***Glycine max*** **.**
(XLSX)
