# Supplementary figures and images for: Species-Specific Expansion and Molecular Evolution of the 3-hydroxy-3-methylglutaryl Coenzyme A Reductase (HMGR) Gene Family in Plants
Source: PLoS One. 2014 Apr 10;9(4):e94172. doi: 10.1371/journal.pone.0094172 (PMC3983158; doi:10.1371/journal.pone.0094172)

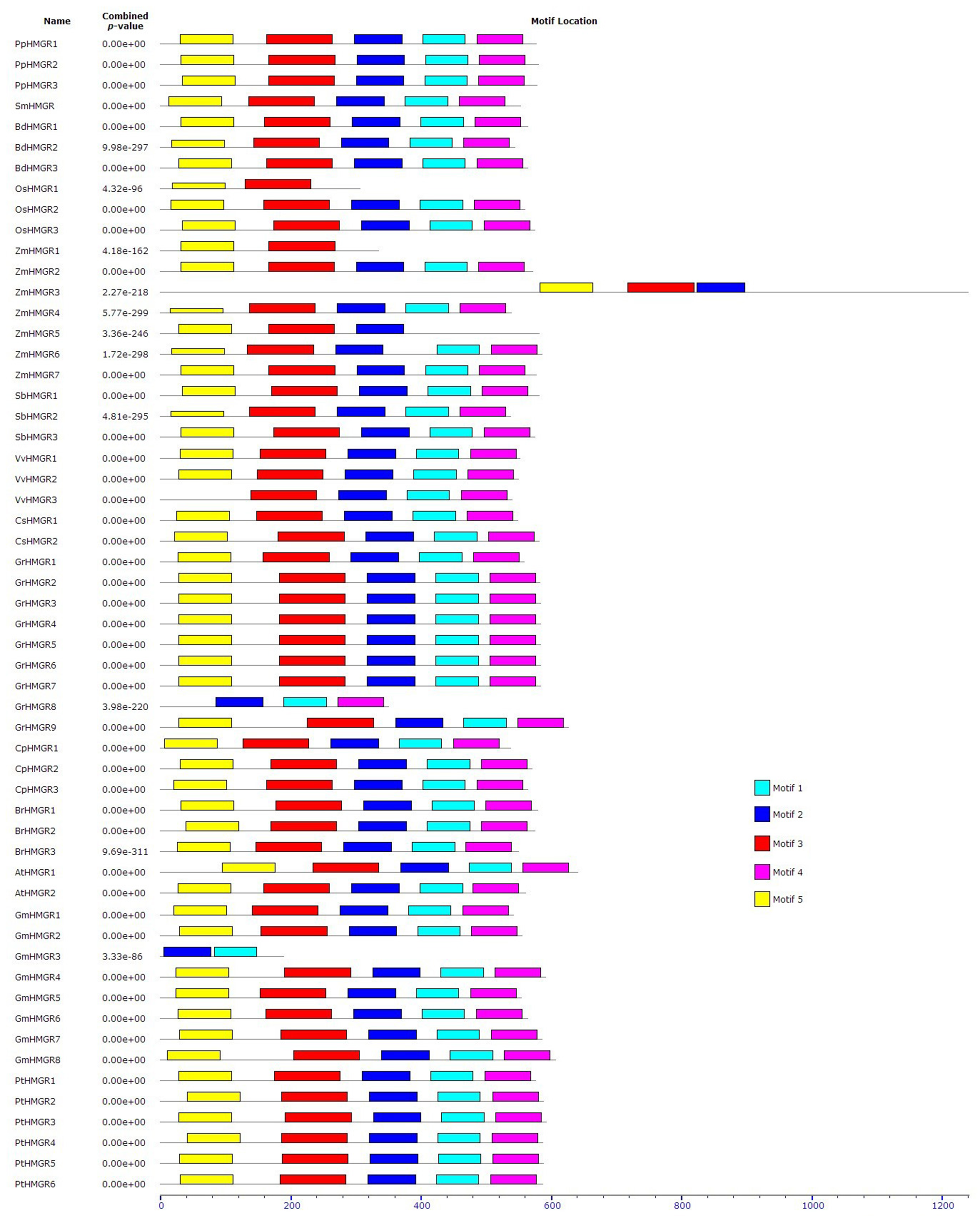

Supplement: Figure S1 — Distribution of conserved motifs in plant HMGR proteins identified using the MEME search tool. Different motifs are indicated by different colors numbered 1-5. The names of all members of HMGR genes and combined p-values are shown on the left side of the figure, and the positions and sizes of motifs are indicated at the bottom of the figure. The motif 5 is a region including two transmembrane helices. The motif 3, 2, 1 and 4 are located in the catalytic domain of HMGR proteins. Moreover, the N-terminus of the motif 3 is at the start position of catalytic domain in each HMGR protein. (TIF) [file pone.0094172.s001.tif]

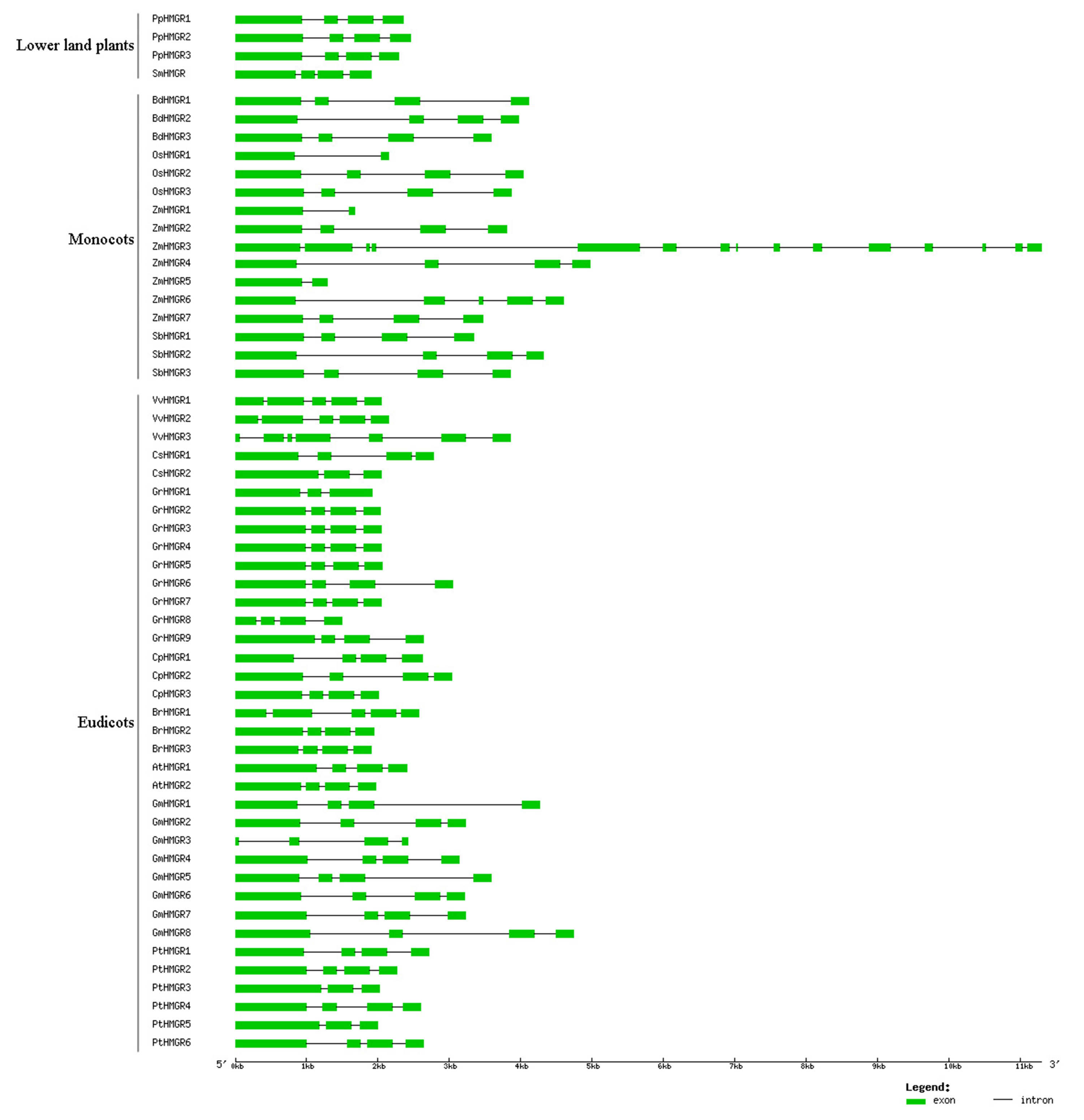

Supplement: Figure S2 — Exon/intron organization of plant HMGR genes. Exons are represented by green boxes and introns by black lines. Names of all plant HMGR genes from different lineages are shown on the left side of the figure. (TIF) [file pone.0094172.s002.tif]
